# Mediastinal Pseudocyst: Varied Presentations and Management—Experience from a Tertiary Referral Care Centre in India

**DOI:** 10.1155/2017/5247626

**Published:** 2017-03-14

**Authors:** Durairaj Segamalai, Abdul Rehman Abdul Jameel, Naveen Kannan, Amudhan Anbalagan, Benet Duraisamy, Prabhakaran Raju, Kannan Devy Gounder

**Affiliations:** Institute of Surgical Gastroenterology, Madras Medical College, Chennai 600003, India

## Abstract

Pseudocysts are a recognised complication following acute or chronic pancreatitis. Usually located in peripancreatic areas, they have also been reported to occur in atypical regions like liver, pelvis, spleen, and mediastinum. Mediastinal pseudocysts are a rare entity and present with myriad of symptoms due to their unique location. They are a clinical challenge to diagnose and manage. In this paper, we describe the clinical and radiological characteristics of mediastinal pseudocysts in 7 of our patients, as well as our experience in managing these patients along with their clinical outcome.

## 1. Introduction

Pancreatic pseudocysts are common findings in patients with acute or chronic pancreatitis, usually located in peripancreatic areas. Mediastinal pseudocyst is rare and often reported as case reports, exact incidence being unknown [1]. Pancreatic ductal disruption due to inflammatory injury leads to leakage of amylase rich pancreatic secretions along the paths of least resistance. Posterior disruptions can lead to thoracopancreatic fistulae while anterior disruptions produce pancreatic ascites. Thoracopancreatic fistulas are divided into four types based on the termination site of the fistula: pancreaticopleural, mediastinal pseudocyst, pancreaticobronchial, and pancreaticopericardial. Mediastinal pseudocyst by way of its unique location can present with myriad symptoms like dysphagia, chest pain, or palpitations and in extreme cases pericardial effusion, tamponade, and respiratory distress [2]. It can be a diagnostic and therapeutic challenge. High index of suspicion is often needed in diagnosing this entity. Pancreatic ductal morphology and its communication with the pseudocyst hold the key for successful management. We present our experience in managing mediastinal pseudocysts.

## 2. Materials and Methods

This study is a retrospective analysis of patients diagnosed to have mediastinal pseudocyst between Jan 2010 and March 2016 at our Institute. Our Institute is a high volume tertiary referral care centre in India, where more than 200 pancreatic surgeries are performed annually for various benign and malignant disorders. We reviewed clinical records and imaging database and were able to identify 7 patients with mediastinal pseudocyst. Thorough analysis regarding their clinical symptomatology, etiology of pancreatitis, radiological features, supplementary investigations, management strategy, and their follow-up was done. Basic laboratory investigation including serum amylase, lipase, and c-reactive protein was done. All patients underwent oesophagogastroduodenoscopy (OGD), ultrasound (USG) with portal Doppler, contrast enhanced computed tomography (CT) scan of abdomen and chest, and other investigations like echocardiogram, barium swallow, and magnetic resonance cholangiopancreatography (MRCP) as required. Patient presenting with ascites or pleural effusion underwent aspiration and biochemical fluid analysis. Patients who had dysphagia were graded as per dysphagia score of Knyrim et al. [3]; grade 0 denoted the ability to eat a normal diet; 1, the ability to eat some solid food; 2, the ability to eat semisolids only; 3, the ability to swallow liquids only; and grade 4 referred to complete dysphagia. Subsequent to the definitive intervention, the patients were advised to attend the follow-up clinic at our institution after 3 months or earlier if symptomatic. All patients underwent CT chest/abdomen to document the resolution of pseudocyst and to study the disease activity after 3 months following the primary intervention. Patients were advised to review with us after 6 months thereafter, to document any complications. Follow-up of patients was for a mean of 13 months (range 10 to 18 months). We did not encounter any dropouts in our series.

## 3. Results

The details of the patients are mentioned in Table 1. Of the 7 patients 6 were male and one was female with age ranging from 17 to 40. Our analysis reveals that chronic pancreatitis was present in 6/7 patients with mediastinal pseudocyst, with ethanol being the most common etiological factor causing chronic pancreatitis in 5/6 patients and it was idiopathic in the other patient. Acute necrotising pancreatitis was observed in one patient who had mediastinal pseudocyst.

**Figure 1:**
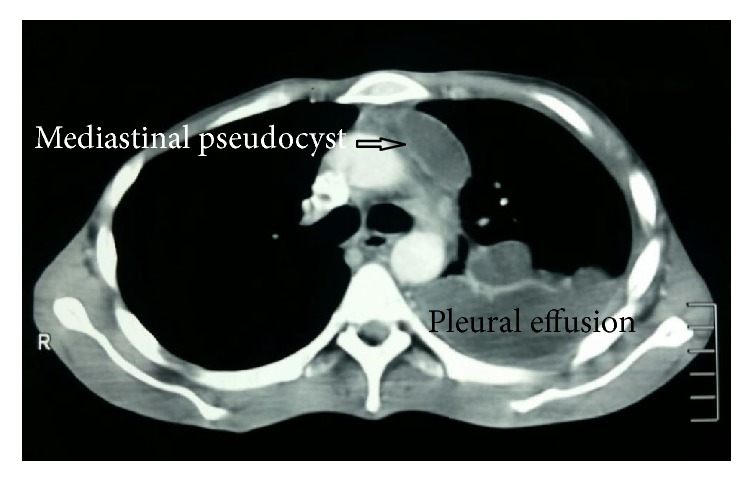
CT chest demonstrating mediastinal pseudocyst of size 3 cm, associated with left pleural effusion.

**Figure 2:**
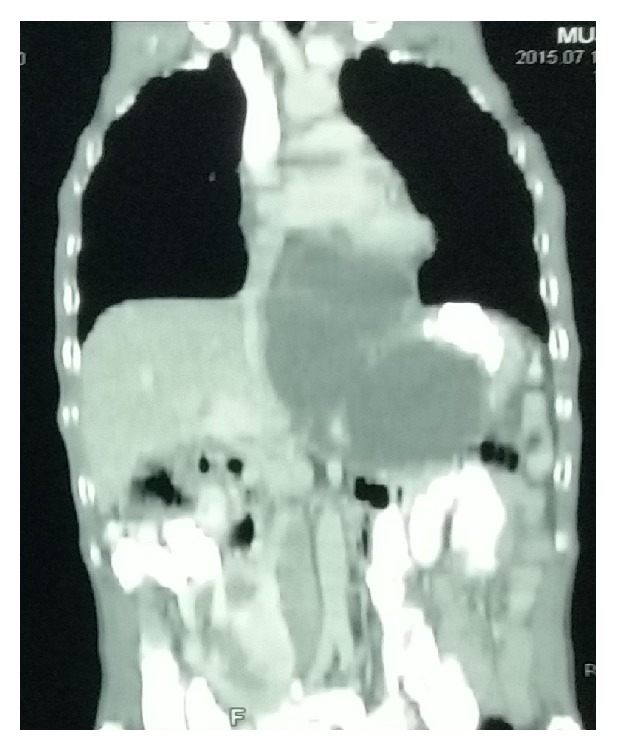
CT chest coronal view showing mediastinal pseudocyst of size 8 cm.

**Figure 3:**
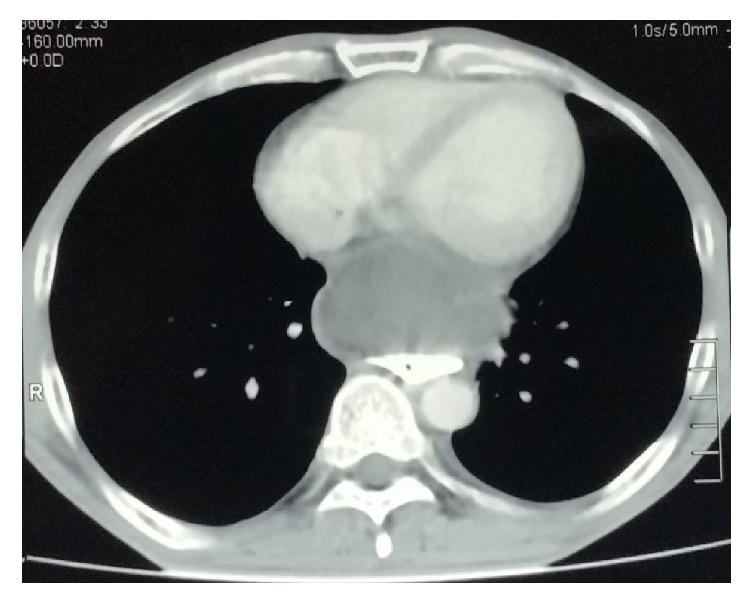
CT chest showing mediastinal pseudocyst compressing the esophagus.

**Figure 4:**
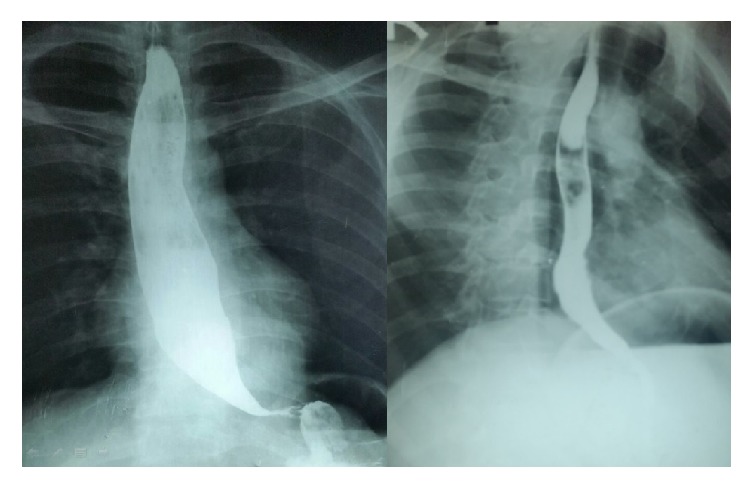
Barium swallow (preop and postop) demonstrating the esophageal dilatation due to mediastinal pseudocyst and resolution of compressive effects following surgery.

**Figure 5:**
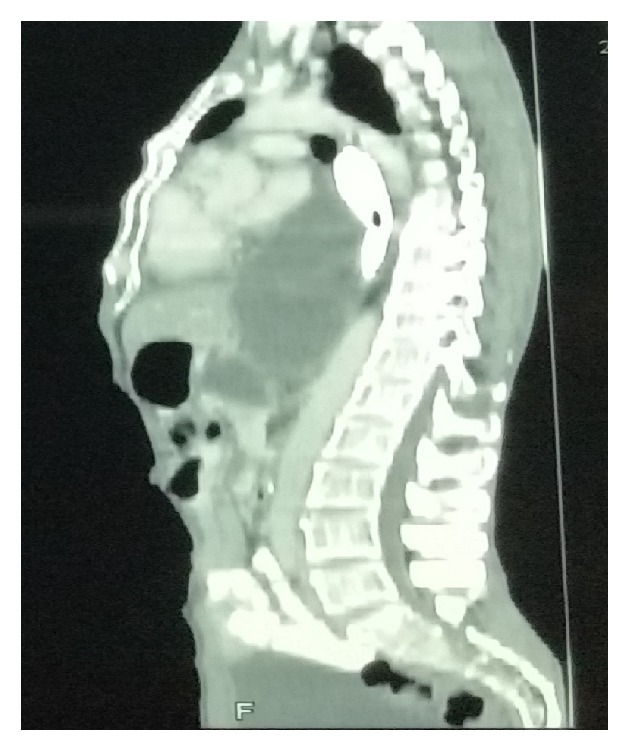
CT chest sagittal view demonstrating the compressive effects on cardiac chambers.

Almost all of the patients had abdominal pain in addition to symptoms attributed to mediastinal pseudocyst like dyspnoea (*n* = 3), dysphagia (*n* = 2), chest pain (*n* = 2), and retrosternal discomfort (*n* = 1). The mean size of the mediastinal pseudocyst encountered in this series was 5.7 cm (ranging from 3 cm to 8 cm) (Figures 1 and 2). All the patients had abdominal pseudocyst. Two patients had dysphagia due to compressive effects of pseudocyst on the esophagus (Figure 3). One of the patients, who had dysphagia with considerable weight loss (>15% in 3 months), was referred to our unit as achalasia cardia on the basis of barium swallow report and he was totally relieved of his symptom following internal drainage (Figure 4). Two patients who presented with dyspnoea had severe left ventricular dysfunction, due to compressive effects on cardiac chambers (Figure 5), which improved following treatment. Pleural effusion was observed in five individuals.

**Figure 6:**
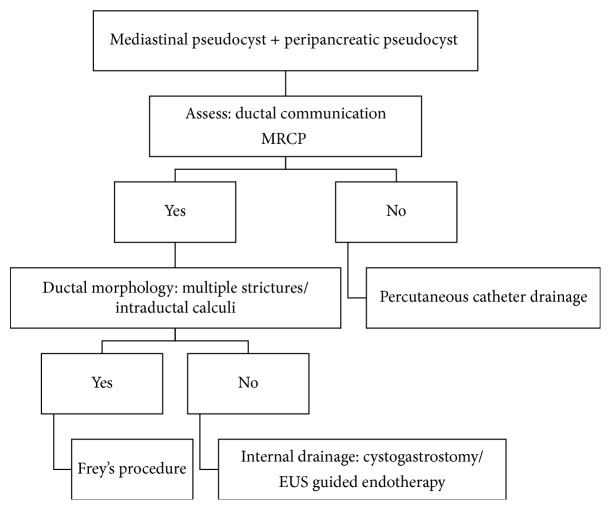
Proposed treatment algorithm for management for symptomatic mediastinal pseudocyst.

**Figure 7:**
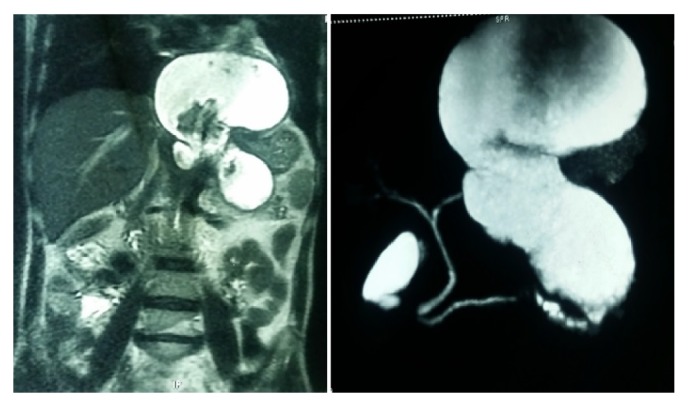
MRI abdomen showing pseudocyst involving tail of pancreas with mediastinal extension and MRCP showing ductal communication of the pseudocyst.

We summarize our proposed treatment algorithm in Figure 6, which is based on our experience in managing such cases. We have noted in our series that mediastinal pseudocyst is invariably associated with peripancreatic pseudocyst and management directed towards the pancreatic pseudocyst leads to resolution of mediastinal component. Pancreatic ductal morphology and its communication (Figure 7) with the pseudocyst as documented by MRCP are the key parameters influencing treatment strategies. In the absence of ductal communication, ultrasound guided insertion of percutaneous catheter drains (PCDs), preferably 10–12 Fr, has been found to be helpful. From our experience, invariably multiple PCDs are required in patients with necrotic or infected collections to ensure complete resolution as they tend to block the catheters and are also not adequately drained by a single catheter. Patients with multiple ductal strictures or intraductal calculi will require a Frey's procedure to adequately drain the entire ductal system. For those with ductal communication with strictures, we advocate internal drainage in the form of cystogastrostomy or cystojejunostomy based on the anatomic proximity of the pseudocyst to the stomach wall. In our series, we performed cystogastrostomy as most of the pseudocysts were related to posterior gastric wall. We encountered pleural effusion in five patients but only two of them required insertion of intercostal drainage tube, in view of symptoms.

Internal drainage could also be performed by endoscopic approach, preferably with endoscopic ultrasound guidance (EUS), if expertise is available. We did not employ endoscopic approach in our series as endoscope could not be negotiated beyond oesophagogastric junction in two patients, while two patients did not require internal drainage and were managed with PCDs. One of our patients had multiple peripancreatic pseudocyst with walled-off pancreatic necrosis, for whom we felt that surgical drainage would offer better results. Endotherapy could not be considered for the patient with multiple ductal stricture associated with intraductal calculi. To summarize, internal drainage in the form of cystogastrostomy was performed in four patients, while Frey's procedure was done in one patient. Two patients were managed with multiple PCDs and intercostal drainage.

Apart from the specific management, patients were generally managed with intravenous fluids, analgesics, antiemetics, and antibiotics being initiated in those with infected pseudocysts. Patients tolerating oral diet were encouraged to do so with low-fat diet, while those with low or no intake were put on nasojejunal feeding and total parenteral nutrition instituted when nasojejunal tube could not be placed. Our unit does not use somatostatin analogues in such patients due to lack of strong evidence in literature.

**Figure 8:**
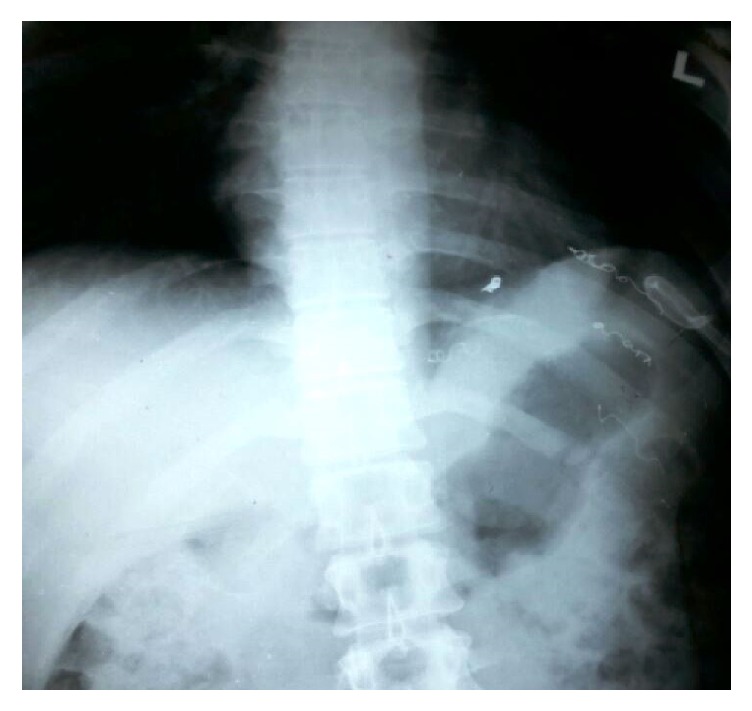
Chest X-ray demonstrating multiple coil embolization done for pseudoaneurysm of left inferior phrenic artery and multiple intercostal arteries. Note the presence of percutaneous placed catheter for drainage of peripancreatic necrosis.

**Figure 9:**
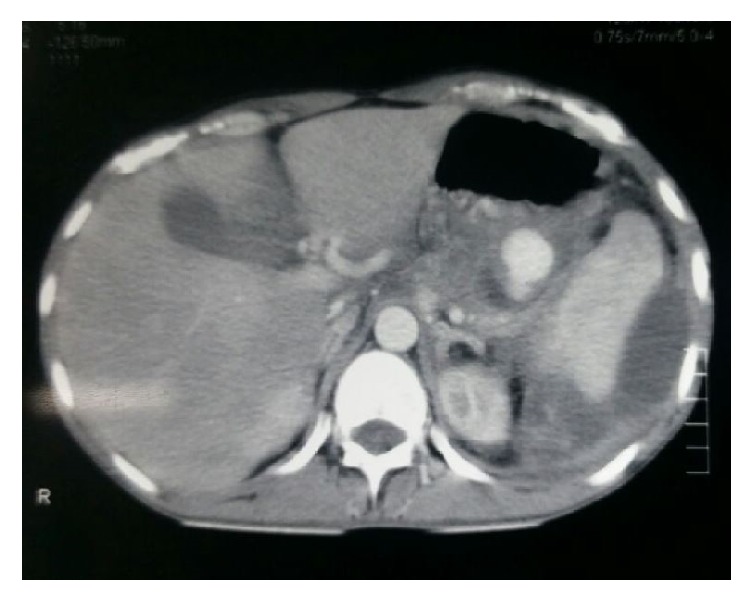
Contrast enhanced CT abdomen showing splenic artery pseudoaneurysm.

**Figure 10:**
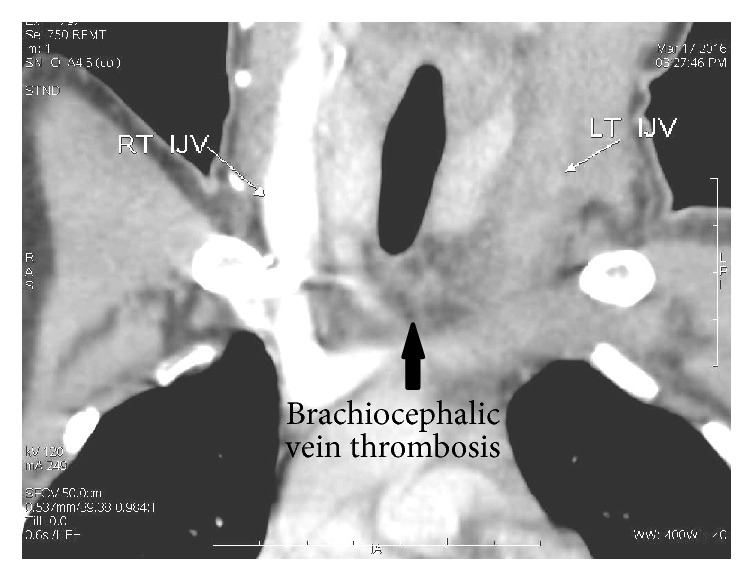
Contrast enhanced CT chest showing nonvisualization of left internal jugular vein and brachiocephalic vein due to thrombosis in a case of mediastinal pseudocyst.

Interestingly, we noted increased incidence of vascular complications in these patients. For the patient with pancreaticopleural fistula, reported with hemoptysis 2 weeks after intercostal drainage, CT-angiogram showed multiple pseudoaneurysm involving inferior phrenic and lower intercostal arteries which was eventually managed with angioembolisation with coils (Figure 8), while another patient with infected pseudocyst developed splenic artery pseudoaneurysm in the follow-up period and required angioembolisation (Figure 9). Mediastinal pseudocyst resulted in left brachiocephalic and left internal jugular vein (IJV) thrombosis (Figure 10) in a patient who was put on anticoagulant therapy for 6 months and subsequently recanalised.

## 4. Discussions

Abdominal complications like pseudocyst and pancreatic necrosis are recognised complications which occur as sequelae to acute or chronic pancreatitis [4]. Mediastinal pseudocyst is a rare complication, usually detected on imaging studies performed for pancreatitis [5]. Presence of inflammation and fibrosis along the traditional peripancreatic spaces creates pathways of lesser resistance to mediastinum to form thoracopancreatic fistulas [6]. Thoracopancreatic fistulas are divided into four types based on the termination site of the fistula: pancreaticopleural, mediastinal pseudocyst, pancreaticobronchial, and pancreaticopericardial [7]. In most cases, the pseudocyst is located in the posterior mediastinum, with entry to the mediastinum via the aortic or esophageal hiatus [4].

Most patients are alcoholics with a clinical history of previous pancreatitis. The presentation is often confusing because of the paucity of clues suggestive of pancreatic disease and the preponderance of pulmonary signs and symptoms. The most common presenting symptoms are chest or abdominal pain and dyspnoea [8]. Due the mediastinal location of the pseudocyst, dysphagia occurs because of compression of the esophagus by the pseudocyst [6]. In our study, two patients had dysphagia. Since the origin of pseudocyst is a ductal disruption in the pancreas, invariably most of our patients had abdominal pain and coexistent abdominal pseudocysts.

Ultrasound is an easily available investigation to diagnose peripancreatic pseudocyst, but it is less helpful in mediastinal pseudocyst owing to its location. Computed tomography (CT) is excellent in defining pancreatic abnormalities and should be the first abdominal imaging study in suspected cases [9]. CT can also comment on the connection between the mediastinal cystic structures and the pancreas. MRI can help in delineating the communication of mediastinal pseudocysts with an abdominal pseudocyst; in addition ductal morphology like disruption, communication with pseudocyst, stricture, and dilatation are best defined by magnetic resonance cholangiopancreatography (MRCP) [5]. Endoscopic ultrasound (EUS) is an important diagnostic tool for the evaluation of mediastinal mass and cysts, and it can help in planning the optimal therapy and allow EUS guided aspiration and drainage of the cysts but is limited by availability of equipment and expertise [10].

The management of mediastinal pseudocysts depends on the clinical symptomatology, underlying etiology, ductal anatomy, size of the pseudocyst, and availability of expertise. Spontaneous resolution of mediastinal pseudocyst with conservative management is a rare event [11]. Endoscopic procedures have significantly influenced the management of mediastinal pseudocysts. EUS assisted endoscopic drainage through either a transoesophageal [1, 12] or transgastric approach has been described with immediate technical success in 90–95% of patients and long term success in 85–90% patients [13]. Endoscopic retrograde cholangiopancreatogram (ERCP) has been employed for transpapillary stenting of the pancreatic duct and few reports have described successful resolution of mediastinal pseudocysts with transpapillary stenting alone [12, 14]. ERCP carries with it its own set of complications, including pancreatitis, haemorrhage, duodenal perforation, and cholangitis. Complications of EUS guided cyst aspiration include perforation of the oesophagus, infection, and stricture formation [12]. The general complication rate of endoscopic management procedures for pancreatic pseudocysts is approximately 5% and pancreatic pseudocysts recur in approximately 15% of patients [15].

Surgical treatment has often been used for therapeutic management of patients with mediastinal pseudocyst and these can vary from pancreatic resections to external or internal drainage. In our study the majority of the patients were treated with surgical internal drainage like cystogastrostomy in four patients and Frey's pancreaticojejunostomy in one patient. Successful resolution of mediastinal pseudocysts with less invasive procedures, such as combined laparoendoscopic or thoracoscopic approaches, has also been reported [16]. Interventional radiological procedures are often useful adjunct in management of complications following pancreatitis and are also useful in managing mediastinal pseudocyst. The same principle of percutaneous catheter guided drainage (step-up approach) [16] for peripancreatic collections/necrosis is applicable here as well. Drainage of the abdominal pseudocyst/necrotic collections resulted in resolution of mediastinal pseudocyst as observed in two of our patients.

Vascular complication in the background of chronic pancreatitis is a rarely observed complication but is potentially life threatening [17]. It could range from bleeding from visceral artery pseudoaneurysm to thrombosis of peripancreatic veins as encountered in our series [18, 19]. We are probably the first to report brachiocephalic vein thrombosis, due to mediastinal pseudocyst. The development of thrombosis is due to local, prothrombotic, inflammatory changes in the vascular endothelium and by extrinsic compression due to pseudocysts [20]. We would like to highlight the fact that development of a complication following pancreatitis creates an opportune environment for another complication.

There have been multiple reports of mediastinal cysts causing cardiac failure due to their compressive effects on the cardiac chambers [21]. It is interesting to note that two patients in our series had left ventricular failure with decreased ejection fraction which recovered after resolution of the cyst following treatment.

## 5. Conclusion

Mediastinal pseudocyst is a rare complication following acute or chronic pancreatitis, which should be kept in mind in a patient presenting with atypical symptoms. Thorough evaluation guides to the optimal treatment required. Traditional surgical drainage which treats the underlying pancreatic disease, including ductal decompression or pseudocyst decompression, is effective. Radiological interventions are a useful adjunct to surgical management. EUS guided approach, when feasible, is gaining more favour with growing expertise and advancing technology in endoscopic adjuncts. Mediastinal pseudocysts often require multiple expertise and should be managed in centers with such expertise.

## Figures and Tables

**Table 1:** The table shows patient demographics, presentation, clinical and radiological finding, management, and follow-up.

S. number	Age/sex	Etiology	Acute/chronic	Presenting symptoms	Size of mediastinal pseudocyst	Presence of abdominal pseudocyst	Associated complications	Management	Follow-up
1	40/M	Ethanol	Chronic	Grade 3 dysphagia^1^ Weight loss Abdominal pain	5 cm	Yes Communicating with mediastinal pseudocyst	Severe OG^2^ junction narrowing in barium swallow Endoscopy could not negotiate beyond oesophagogastric junction	Open cystogastrostomy	Dysphagia relieved Postop barium swallow: normal Gained weight

2	29/M	Ethanol	Acute	Dsypnoea Pain abdomen Hemoptysis, 2 weeks following intercostal drainage	8 cm	Yes 8 × 8 cm infected necrosis involving body & tail of pancreas	Pancreaticopleural fistula 40% necrosis of pancreatic body & tail Echocardiogram: severe left ventricular dysfunction EF^3^ 37% Inferior phrenic & intercostal artery pseudoaneurysms	Infected necrosis was managed with 2 percutaneous drainage catheters inserted with ultrasound guidance in left subphrenic & perinephric region Intercostal drainage Angioembolisation of pseudoaneurysms	Follow-up Complete resolution of pseudocyst after 2 months Echocardiogram: EF 70%

3	31/M	Ethanol	Chronic	Abdominal pain Dsypnoea	4.5 cm	Yes Infected pseudocyst 8 × 8 cm: head and body of pancreas	Left pleural effusion Pancreatic ascites	Three PCDs inserted in left subphrenic, left perinephric region and pelvis Left intercostal drainage	2 months later, he developed splenic artery pseudoaneurysm: angioembolisation done

4	36/M	Ethanol	Chronic	Chest pain Early satiety Abdominal pain	3 cm	Yes 8 × 6 cm pseudocyst in head of pancreas	Nil	Open cystogastrostomy	Complete resolution of pseudocyst after 1 week

5	39/M	Ethanol	Chronic	Retrosternal discomfort Dsypnoea Pain abdomen	8 cm	Yes Multiple peripancreatic pseudocyst	Left pleural effusion/walled-off pancreatic necrosis Echocardiogram: severe left ventricular dysfunction EF 40%	Open cystogastrostomy, open necrosectomy, external drainage	Pseudocyst Resolved EF improved to 64%

6	33/M	Ethanol	Chronic	Chest pain Dysphagia Abdominal pain	6.5 cm	Yes. Multiple peripancreatic pseudocyst	Bilateral pleural effusion Extrinsic compression over esophagus from 34–38 cm	Open cystogastrostomy	Resolutions of symptoms

7	17/F	Idiopathic	Chronic	Abdominal pain Left sided neck pain and edema	4 cm	Yes Pseudocyst 2 × 2 cm head of pancreas Multiple parenchymal and ductal calculi	Left pleural effusion Left IJV^4^/brachiocephalic vein thrombosis	Frey's procedure	Thrombus recanalised after 6 months of anticoagulant therapy

^1^Dysphagia score of Knyrim et al.

^2^Oesophagogastric.

^3^Ejection fraction.

^4^Internal jugular vein.

## Additional Points

*Core Tip*. Mediastinal pseudocysts are a rare entity, most of the them published as case reports; we present a series of 7 cases. The highlighting point is the varied symptomatology of the patients. The various management strategies that were employed in these were tailored to the individual patients. Interesting complications and outcomes are also recorded. This paper might serve as guide to surgeons who encounter such rare cases in their clinical practice.
